# A Functional Variant rs1820453 in *YAP1* and Breast Cancer Risk in Chinese Population

**DOI:** 10.1371/journal.pone.0079056

**Published:** 2013-11-01

**Authors:** Wei Chen, Wei Wang, Beibei Zhu, Hui Guo, Yu Sun, Jie Ming, Na Shen, Zhi Li, Zhenling Wang, Lifeng Liu, Bingxi Cai, Jiayu Duan, Jiaoyuan Li, Cheng Liu, Rong Zhong, Weiguo Hu, Tao Huang, Xiaoping Miao

**Affiliations:** 1 State Key Laboratory of Environment Health ( Incubation), Ministry of Education Key Laboratory of Environment & Health, Ministry of Environmental Protection Key Laboratory of Environment and Health (Wuhan), and Department of Epidemiology and Biostatistics, School of Public Health, Tongji Medical College, Huazhong University of Science and Technology, Wuhan, China; 2 Department of Breast and Thyroid Surgery, Union Hospital, Tongji Medical College, Huazhong University of Science and Technology, Wuhan, China; 3 Department of Breast Surgery, Hubei Cancer Hospital, Wuhan, China; 4 Department of Oncology, Renmin Hospital of Wuhan University, Wuhan, China; University of North Carolina School of Medicine, United States of America

## Abstract

**Background:**

To investigate the association between rs1820453 which located in the promoter region of yes-associated protein 1 (YAP1) gene and breast cancer (BC) risk.

**Method and Findings:**

We conducted a hospital-based case-control study including a total of 480 BC cases and 545 cancer-free controls in Chinese population. Then the expression quantitative trait locus (e-QTL) analysis was performed to explore the possible function of rs1820453 to the *YAP1* gene expression. The association between rs1820453 and BC risk was significantly identified with the odds ratio (OR) was 1.27 (95 % conﬁdence interval (CI) =1.03-1.57) under allelic model when adjusted by age and menopausal status. In addition, the correlation analysis of rs1820453 and *YAP1* expression level found that this variant was significantly associated with the gene expression in Chinese population. When compared with level of mRNA expression of the AA genotype (6.011±0.046), the mRNA expression level in CC genotype (5.903±0.026) was statistically lower (*P*=0.024).

**Conclusion:**

The results from this study suggested that rs1820453 A>C change may affect the gene expression and contribute to the risk of developing BC in Chinese population though larger sample-size studies along with functional experiments were anticipated to warrant the results.

## Introduction

Breast Cancer (BC) as the most commonly diagnosed cancer among female in the world has been one of the most important global public health problem with much attention has been paid on it [1]. Much effort has been executed to explore the association between BC risk and genetic variants as genetic factor was considered to be a important risk factor for BC susceptibility [2-4]. Though, population-based studies have estimated that high penetrance mutations of *BRCA1* and *BRCA2* account for about 15% of the familial aggregation of BC [5]. However, genetic variants that contribute to the susceptibility of developing sporadic BC are still unclear [6].

Yes-associated protein 1 gene (*YAP1*) located at 11q22, a site of frequent loss of heterozygosity in sporadic BC [7,8] have been indicated that may act as a tumor suppressor [9]. The protein *YAP1* gene encoded was a transcriptional coactivator which was important in P73-dependent apoptosis [10,11]. It has been reported that YAP1 interact with p53-binding protein-2 (ASPP2) which was an important regulator of the apoptotic activity of p53 [12,13]. 

Several studies have shown that YAP1 may bind to the p53 family member p73 and was critical for the DNA damage induced in BC cells and some other types of neuronal apoptosis [11,14,15]. YAP1 loss by siRNA may protect BC cells from DNA damage-mediated apoptosis which then may promote the developing of tumor. Furthermore, YAP1 has also been reported to stabilize the P73 protein of the post-translation stage [16,17]. 

It was demonstrated that YAP1 protein expression was decreased or lost in breast cancers [9]. In addition, Yuan M et al, have found that BC cells with YAP1 silencing show increased migration and invasion which then enhanced tumor growth. 

Intriguingly, a recent research identified a *YAP1* variant, rs1820453, was associated with survival of small-cell lung cancer patients and the variant A>C change created a transcriptional factor binding site which then resulted in the down regulation of *YAP1* expression [18]. Since YAP1 protein expression was frequently found to be decreased in breast cancers and rs1820453 A>C may down-regulate the *YAP1* expression; we have interesting to know whether this variant is associated with BC risk. Therefore, we carried out a hospital-based case-control study to investigate the association between rs1820453 and BC risk. 

## Materials and Methods

### Ethics Statement

After written informed consent was obtained, 2 ml peripheral venous blood sample and the characteristic data were collected from each participant at the recruitment. The age and menopausal status of each subjects and the ER and PR status of each patients were collected. However, due to the poor memory of some participants, the characteristics of age of menarche and family history, which were originally expected to be collected, were not completed finally. In addition, the blood samples were stored in the -80°C refrigerator before the DNA was extracted and the samples were transformed by the cold chain. This study was approved by the ethnics committee of Union Hospital of Huazhong University of Science and Technology and Tongji Medical College, Huazhong University of Science and Technology.

### Study subjects

First, 506 cases and 576 cancer-free controls were asked to participate in the study. But 18 cases and 21 controls were excluded because of their declining to the research. Therefore, 488 cases and 555 controls were included for genotyping. All subjects were genetically unrelated Chinese Han women living in Wuhan city and surrounding regions. Patients were consecutively recruited between June 2009 and December 2011 at the Union Hospital of Huazhong University of Science and Technology, Wuhan, China. All cases were histopathologically confirmed without any previous radiotherapy and chemotherapy before they were included in this study; and there is no restriction about age and type of BC or disease stage. Controls were cancer-free individuals randomly selected among the health check-up persons at the same hospital in the same time period as cases were enrolled. In addition, the controls were frequency matched to the cases for age (± 5 years). 

### Genotyping

Genomic DNA was extracted from the whole blood sample of all participants using the RelaxGene Blood System DP319-02 (Tiangen, Beijing, China) according to the manufacturer’s directions. The quantity and quality of DNA　was assessed by the NanoDrop 2000 spectrophotometer. The genotype of rs1820453 was determined by the TaqMan SNP Genotyping Assay (Applied Biosystems, Foster city, CA) using the 7900HT Fast Real-Time PCR System (Applied Biosystems, Foster city, CA). To ensure quality control, 5% duplicated samples were randomly selected to evaluate the reproducibility and with 100% concordance. Moreover, genotyping was performed without knowledge of case or control status. The call rate of genotyping was 98.3 %, and 480 patients and 545 controls were finally included for subsequent statistical analyses. 

### Statistical analyses

Hardy-Weinberg equilibrium (HWE) for rs1820453 was evaluated by goodness-of-fit χ^2^ test for genotypes in the control group. Difference in distribution of demographic characteristics between cases and controls were evaluated by χ^2^ test and *t* test where appropriate. The association between BC risk and rs1820453 was assessed by the odds ratio (OR) along with 95 % confidence interval (95 % CI) using unconditional multivariate logistic regression analysis with adjustment for age and menopausal status. All statistical analyses were performed using the SPSS 12.0 software with two-sided *P* value less than 0.05 was considered to be statistical significance. 

### The genotype and mRNA expression data analysis from HapMap database

The genotype-phenotype analysis was carried out to explore the possible function of rs1820453 using the SNPexp database which was available online (http://app3.titan.uio.no/biotools/help.php?app=snpexp) [19] after significant association of rs1820453 and BC risk was found. The genotype data were from the HapMap phase ii release 23 data set including 270 individuals from 4 populations. Among the 270 individuals, 45 were unrelated Chinese Han population from Beijing (CHB), 45 were Japanese in Tokyo (JPT), 90 were Yoruba in Ibadan, Nigeria (YRI) and 90 were Utah persons from northern and western Europe (CEU). The mRNA expression data were from EBV-transformed B lymphoblastoid cell lines from the same populations.

## Results

### Results of case-control study

#### Characteristics of study subjects

The distributions of demographic characteristics of the participants were presented in Table 1. A total of 1025 subjects including 480 cases and 545 controls were analyzed in the current study. The mean age was 48.30 years (±9.91) and 48.87 years (±12.51) for case and control group, respectively; and no significant difference (*P*= 0.417) were identified. In addition, there were more postmenopausal persons in controls but no significant distribution difference (*P*= 0.094) of menopausal status between cases and controls was found. 

**Table 1:** The characteristics of the case-control study population.

Variable	Case (480) No (%)	Control (545) No (%)	*P*
Age	48.30±9.91	48.87±12.51	0.417^a^
Menopause			
No	232 (48.3)	235 (43.1)	0.094^b^
Yes	248 (51.7)	310 (56.9)	
Estrogen Receptor(ER)			
Positive	278 (57.9)		
Negative	167 (34.8)		
Missing	35 (7.3)		
Progesterone Receptor(PR)			
Positive	247 (51.5)		
Negative	198 (41.3)		
Missing	35 (7.3)		

a *P* value was calculated by *t* test

b *P* value was calculated by χ^2^ test

#### Association analysis

The genotypes distribution of rs1820453 for cases and controls and the association between this variant and BC risk were summarized in Table 2. The genotypes in control group were in agreement with the HWE (*P*= 0.942). When considered the A allele as reference, the C allele was significantly associated with increased BC risk with the OR=1.27 (95 % CI=1.03-1.57) after adjusted by age and menopausal status under logistical regression analyses. The persons who carried the CC genotype had significantly increased BC risk when compared to those who carried AA genotype (*P*= 0.009; OR=2.05, 95 % CI=1.19-3.53). Moreover, the significant association between rs1820453 and BC risk were also identified in recessive and additive models with the OR of 1.98 (95 % CI=1.16-3.38) and 1.26 (95 % CI=1.03-1.54), respectively. When classified BC cases according to the Estrogen Receptor (ER) and Progesterone Receptor (PR) status, significant association of BC risk and rs1820453 was only remained in ER-PR- specified BC subgroup but not in ER+/PR+ subgroup (*P* =0.004, OR=1.53, 95 % CI=1.14-2.03 and *P* =0.191, OR=1.18, 95 % CI=0.92-1.50 under allelic model for ER-PR- subgroup and ER+/PR+ subgroup, respectively).

**Table 2:** The association between rs1820453 and BC risk in Chinese population.

*YAP1*-rs1820453	Case (480) No (%)	Control (545) No (%)	*P* ^b^	OR^a^ (95 % CI)
AA	282 (58.8)	345 (63.3)		
AC	160 (33.3)	177 (32.5)	0.455	1.11 (0.85-1.44)
CC	38 (7.9)	23 (4.2)	0.009	2.05 (1.19-3.53)
C/A			0.023	1.27 (1.03-1.57)
Dominant model			0.130	1.22 (0.94-1.56)
Recessive model			0.012	1.98 (1.16-3.38)
Additive model			0.027	1.26 (1.03-1.54)

a adjusted by age and menopause

b all statistical tests were two-sided and *P* <0.05 was used as the criterion of statistical significance

### Results of the Genotype and mRNA Expression Analysis From the HapMap Database

**Figure 1:**
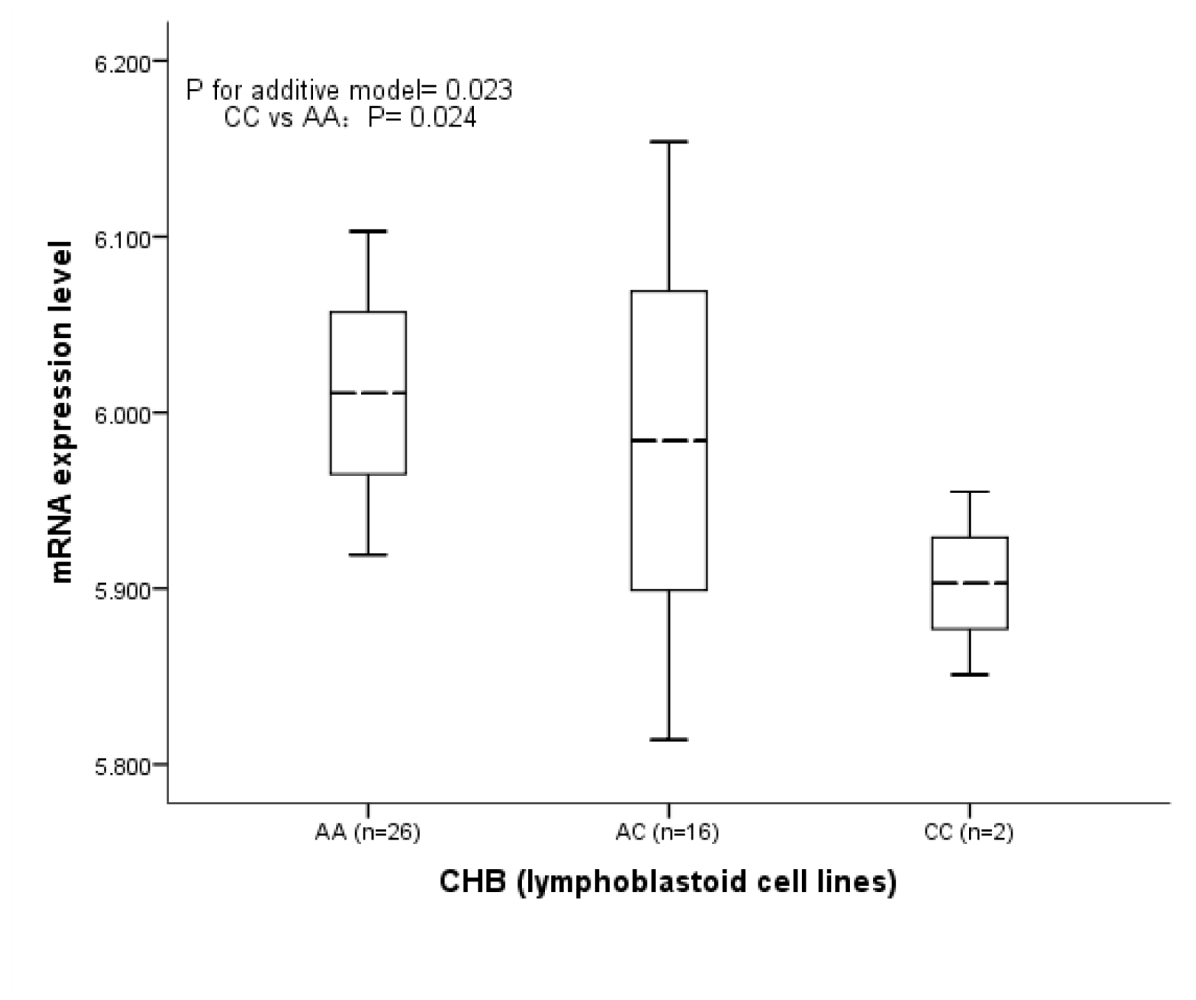
The mRNA expression level of *YAP1* gene in unrelated CHB.

**Figure 2:**
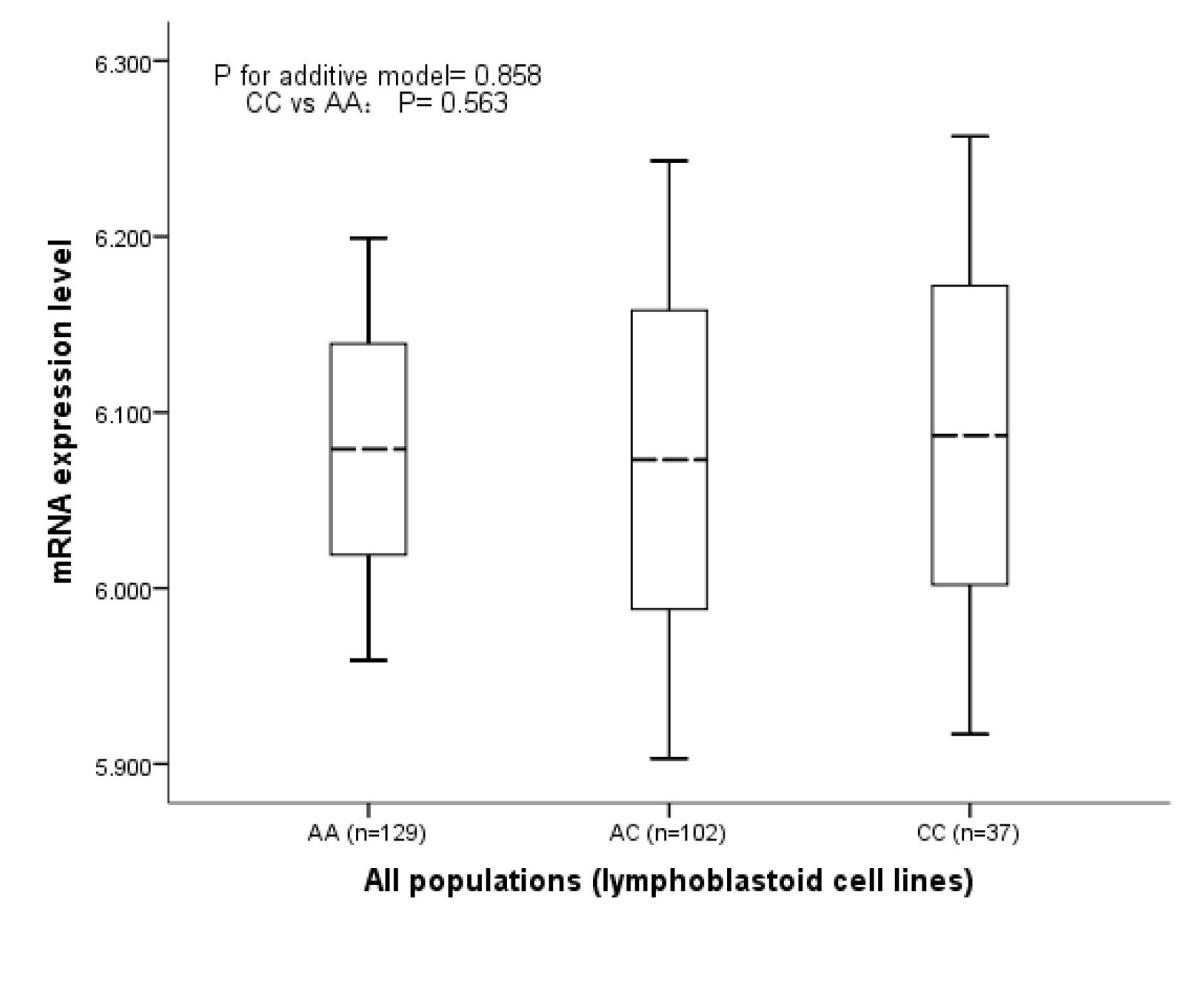
The mRNA expression level of *YAP1* gene in all four populations of the HapMap cell lines.

The correlation analysis of rs1820453 and *YAP1* mRNA expression in 44 (1 missing) unrelated Chinese Han population found that when compared with the levels of mRNA expression in 26 cell lines with rs1820453 AA genotype (6.011±0.046), 2 cell lines with the CC genotype had statistically lower levels of mRNA expression (5.903±0.026, *P*= 0.024) (Figure 1). However, we did not identify significant association of *YAP1* mRNA expression and rs1820453 in the 270 all population HapMap lymphoblastoid cell lines with the mRNA expression levels in 129 cell lines with AA genotype, 102 cell lines with AC genotype and 37 cell lines with CC genotype were 6.079±0.060, 6.073±0.085, and 6.087±0.085; respectively (Figure 2). The all results suggested that rs1820453 A>C change may be the risk factor for BC susceptibility in Chinese through influencing the *YAP1* gene expression. 

## Discussion

In the present case-control study consisted of 480 cases and 545 controls, we identified that rs1820453 A>C change significantly associated with increased BC risk in Chinese population with the OR of 1.27 under the allelic model (*P*= 0.023). Then the genotype-phenotype correlation analysis of rs1820453 and *YAP1* gene mRNA expression level found that the mRNA expression level was significant lower in cell lines with CC genotype than the cell lines with the AA genotype in Chinese population (*P*= 0.024). But it was worth noting that the association of rs1820453 A>C change and *YAP1* gene mRNA expression was not significantly identified in the all four populations (*P* for additive model= 0.858). All of these suggested that rs1820453 may be the risk factor for BC through influencing the activity of *YAP1* gene in Chinese but not in other populations. Certainly, more population-based studies in other ethnic populations are anticipated to further explore the association of rs1820453 and BC risk. 

The protein YAP1 encoded by *YAP1* gene as a transcriptional coactivator played an important role in the P73-driven apoptosis pathway. It has been reported that the function of P73 was critical for cellular responding to the cytotoxic chemotherapy and the P73 level in tumor cells reduced by siRNA or genetic mutations may lead to strong reduction of apoptosis induced by DNA damage [11,20]. On the other hand, YAP1 as the downstream apoptotic gene among the P73- dependent apoptotic signaling was crucial in stabilizing and enhancing P73 activity. Therefore, the variants in *YAP1* gene which may result in the change of YAP1 activity may finally impact the DNA-damage induced apoptosis pathway. 

The rs1820453 located in the promoter region of *YAP1* gene and the variant A>C change may cause down regulation of YAP1 which in turn might weaken the P73-dependent apoptosis of cancer cells and suppress the chemotherapy-induced cancer cell death. All of which then resulted in faster cancer progression. The gene report experiment from Wu C et al, found that rs1820453 A>C change may affect *YAP1* promoter activity; and the rs1820453 A-containing promoter had higher transcriptional activity. The cells containing rs1820453 A allele drove a significantly higher gene expression when compared to the cells containing rs1820453 C allele [18]. 

In addition to the interaction with P73-driven tumor suppression pathway, present study had found that YAP1 displayed as a protein that integrate another RASSF1A-driven tumor suppressor pathway with the P73 pathway [15]. 

Though we first identified that rs1820453 was significantly associated with increased BC risk in the current study; some limitations also should be acknowledged. First, this was a hospital-based case-control study which may bring in selection bias during the participants recruitment. Moreover, the sample size of this study was relatively small. Therefore, it would be important to confirm the results in larger and prospective studies. In addition, environmental risk factors were also important in the developing of BC. However, we did not analysis the environmental effect or the gene-environment interaction effect due to the lacking of information on exposure of environmental risk factors. And the estimated effect of rs1820453 on BC risk was not adjusted by some confounding characteristics such as age of menarche and family history owing to the uncompleted collection of the relevant information. Furthermore, though the e-QTL analysis used the HapMap data identified significant association between the mRNA expression level of YAP1 and rs1820453 genotypes, the sample size was relatively small, especially the CC genotypes in Chinese was just 2. Meanwhile, we did not perform more functional experiments to further support the results because of the deficiency of samples.

In conclusion, our case-control study and e-QTL analysis identified rs1820453 was associated with BC risk. The results provided a basic insight of this variant contribute to the susceptibility of BC though functional analyses and larger sample size studies were needed to warrant the results.
